# Analysis of Density Distribution in a Cylindrical Specimen under Compaction Using the Example of Dry Ice

**DOI:** 10.3390/ma17112658

**Published:** 2024-05-31

**Authors:** Jan Górecki, Maciej Berdychowski, Krzysztof Wałęsa, Boris Kostov

**Affiliations:** 1 Faculty of Mechanical Engineering, Institute of Machine Design, Poznan University of Technology, 60-965 Poznan, Polandkrzysztof.walesa@put.poznan.pl (K.W.); 2 Department of Thermodynamics, Hydraulic and Ecology, University of Ruse, Studentska 8, 7017 Ruse, Bulgaria

**Keywords:** density distribution, FEM, compaction, dry ice, carbon dioxide

## Abstract

When dealing with processes involving the compaction of bulk materials, very often the quality of the product is determined based on density measurements. Methods used in the industry do not produce compacted materials with high degrees of homogeneity. As a result, the quality of the resulting product, interpreted as its density, varies over the cross-section of the product. In this article, the authors present the results of a numerical study involving the analysis of the density distribution of compacted dry ice during the reciprocating process. The Drucker–Prager/cap model was used in this study, which allowed the change in mechanical properties of the compacted material to be taken into account during the simulation of the process. The diameter, height and density of the cylindrical specimens used in the numerical tests were taken as the variable parameters. Thus, as a result of the testing, the authors could formulate conclusions relating to their impact on the homogeneity of the material.

## 1. Introduction

Compaction is used in many production processes, for instance in the chemical industry [1], pharmaceutical industry [2] or in the production of biofuels [3]. It is described in the literature as an action related to a reduction in the bulk volume of the material due to the removal of the gaseous phase under the influence of applied external stress. The degree of the material’s densification can be determined by a relative coefficient describing the ratio of the difference between its initial and final volume in the process. The indicated method of the estimation of this coefficient is applicable to the compaction of dry materials that do not undergo phase transformation during processing.

As the value of the degree of compaction of the material increases, an increase in the consolidation of the compacted material, understood as an increase in mechanical strength, is observed. This is an effect of interactions occurring at the particle–particle interface [4,5]. Razouki et al. indicated that the quality of the compacted material can be seen by its ability to maintain the stability of its formed shape [6]. The available literature offers a method of indirect assessment of the quality of a compacted product, e.g., by using its density value.

Matsunami et al. indicated that the density value of the material is not uniform throughout its entire volume [7]. As a result, the averaged value of material density determined from the measurement of its mass and volume does not allow an assessment of the cohesion of the agglomerate at its specific points. That is why, due to the non-uniformity of the density distribution within the material, there is a problem in determining the correct factor for the comparison of two identical products, produced, for instance, using different means of production. This finding is confirmed in the literature dealing with studies on the density distribution of materials, among others, materials produced during the densification of powders in the pharmaceutical industry [8] or during the manufacture of layered organic composites [9].

The solution to this problem was proposed in the article by Sinka et al., 2004 for the case of measuring the density distribution inside pharmaceutical tablets. This was made possible through the use of computed tomography, which allows for non-destructive testing, enabling observation of the internal structure of the examined object [10].

The authors observed a non-uniform density distribution (DD) in the end products of the carbon dioxide (DI) pellet extrusion process. This, due to the peculiar properties of DI, significantly affects the possibility of its effective use, e.g., in the cooling process [11]. It results from the unforced phase transition that the material undergoes, i.e., sublimation under normal conditions [12]. As a result of DI densification, the external surface area of the material on which the indicated phase transition is occurring is reduced, which directly affects the speed of the material’s sublimation [13]. DI pellets are stored in insulated containers, where, however, they are not completely protected, e.g., against external forces generated during transport. As a result, the material is damaged in areas with a lower density value, which, in turn, results in an increase in the external surface area of the material and, in consequence, in reduced available time for its use. Therefore, it can be pointed out that, among other things, in the case of DI, the uniformity of DD significantly affects the final quality of the pellet.

No information on the methodology for measuring the density distribution on the surface of a compacted DI section was found by the authors in the available literature. Available methods of use for other materials are not suitable for DI-related testing [14,15]. This is due to the increase in the sublimation rate of the material when in contact with an object with a temperature value higher than that of DI, i.e., 194 K [16]. The use of measuring instruments in an environment with an indicated temperature requires additional calibration and the manufacturers do not guarantee correct indications of the devices.

In light of the above gap of knowledge identified by the authors, this article presents the results of numerical simulations of the DI compaction process. To begin with, simulations of the DI compaction process were performed for cylindrical specimens with different diameters (d) and final heights (h), based on the Drucker–Prager/cap (DPC) [17] model described in an earlier publication. The uniformity of the density distribution was assessed using an averaged coefficient whose value was equal to the absolute value of the difference between the average density of the specimen and the average density of the numerical element in which the measurement was taken.

The research results presented in this article are applicable as a complement to the studies available in the literature, pertaining to the search for geometric parameters of working mechanisms that help reduce power consumption during the DI extrusion process. In the authors’ previous research, the topic of DD uniformity was not addressed, which had a significant effect on the potential of the application of the numerical studies’ results under real industrial conditions.

## 2. Materials and Methods

### 2.1. Material

**Figure 1:**
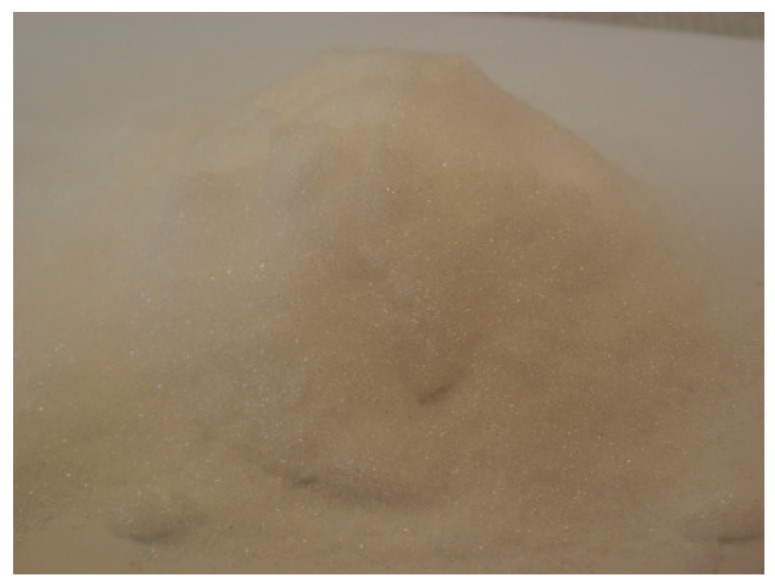
Pulverised dry ice [18].

The study material was pulverised dry ice. It is solid carbon dioxide that has received its common name due to its properties under normal conditions, i.e., at 194 K, and which undergoes a phase transition to the gaseous state [16]. The test material was produced by adiabatic transformation during the rapid expansion of liquid carbon dioxide stored at a pressure of 20 bar. As a result of its expansion to atmospheric pressure, the temperature value of the material was reduced, causing it to change its state of aggregation to a solid form. The dry ice thus obtained has the powdered form, where, as indicated by Liu et al. 2012, the value of the fraction does not exceed 100 μm [18]. The appearance of the material is illustrated in the photograph below (Figure 1).

**Figure 2:**
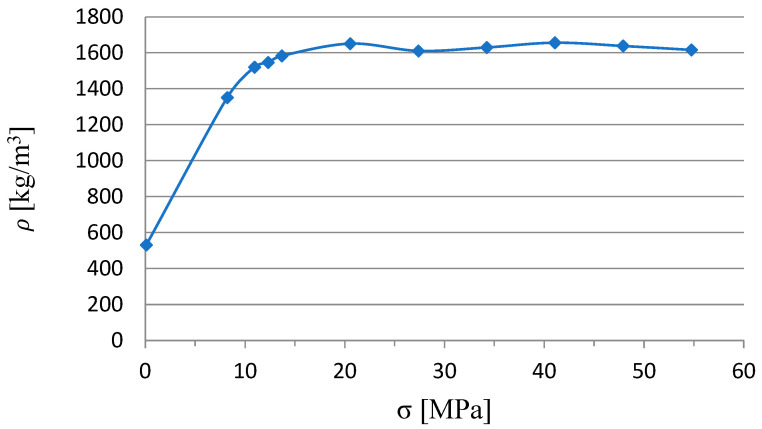
Dry ice density characteristics [12].

In previous studies, the density characteristics of the material were determined, where the bulk density value was 550 kg/m^3^ and the limit value was 1650 kg/m^3^ (Figure 2). Densification of the material to above 1100 kg/m^3^ allows it to be consolidated to such an extent that it is able to give the DI a fixed geometric shape that does not disintegrate due to gravity force [17].

Additionally, in the literature, the authors made available the results of research work on the numerical models developed, where the nature of the change in the values of the material’s mechanical parameters as a function of density, such as Young’s modulus, Poisson’s ratio or coefficient of friction, was determined. For those interested, we recommend the indicated publications [19,20,21].

### 2.2. Numerical Model of DI Compaction Process

**Figure 3:**
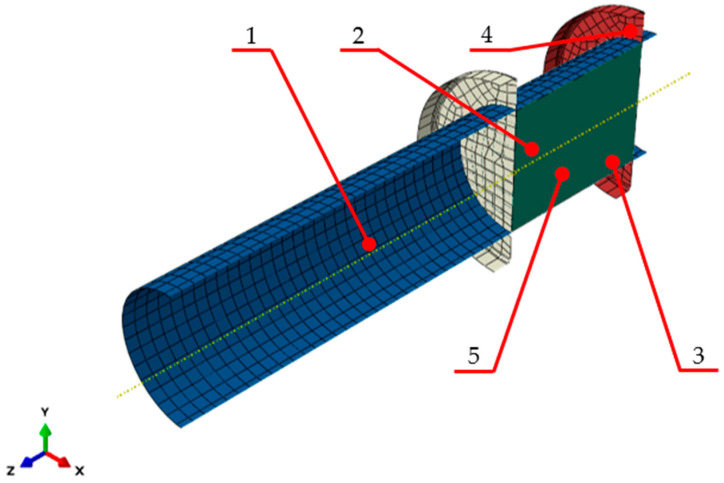
Model used to simulate dry ice compaction in a closed chamber: 1—barrel, 2—ram, 3—extruded dry ice, 4—dead-end disc, 5—force measurement point [19].

Numerical calculations were carried out using the model shown in Figure 3. It is made up of a compression barrel, the material being compacted and two discs: one disc blocking the outlet of the die and one disc acting as the ram. The barrel and the discs were modelled as non-deformable surface objects, i.e., discrete rigid parts. A discrete rigid part is assumed to be rigid and is used in contact analyses to model bodies that cannot deform.

The material that is being compacted was modelled as a deformable object and the Drucker–Prager/cap (DPC) model was used to describe its mechanical properties. The DPC model belongs to the group of phenomenological elastic–plastic models. The DPC comprises three failure surfaces described by a *p*-*q* surface (where *p* is the hydrostatic stress and *q* is the equivalent Mises stress). The model includes a Drucker–Prager failure line defined as *F_S_*, cap surface *F_C_* and a transition surface *F_t_*. Diarra et al. 2017 indicated that the equations describing the model surfaces could be expressed as the following equations [22]:

(1) $$F_{S}=q-p\;tan\beta -d=0$$

(2) $$F_{C}=\sqrt{{\left(p-p_{a}\right)}^{2}+{\left(\frac{Rq}{1+\alpha -\alpha /cos\beta }\right)}^{2}}-R\left(c+p_{a}tan\beta \right)=0$$

(3) $$F_{t}=\sqrt{{\left(p-p_{a}\right)}^{2}+{\left[q-\left(1-\frac{\alpha }{cos\beta }\right)\left(c+p_{a}tan\beta \right)\right]}^{2}}-\alpha \left(d+p_{a}tan\beta \right)=0$$

where *β* is the friction angle, *R* is the eccentricity, *c* is powder cohesion and *p_a_* is an evolution parameter. The α parameter ensures a smooth transition between the cap surface and the shear failure segment.

The barrel and the bottom end disc were immobilised by removing all their degrees of freedom. The top disc acting as the compression ram could only move along the barrel axis with a fixed speed of 5 mm/s. In addition, surface-to-surface interactions/contact between the material being compacted and the other elements making up the model were defined. The value of the friction coefficient *µ* = 0.1 was set in the contact properties.

Since numerous publications [19,20,21] have demonstrated that the mechanical properties of dry ice change with a change in the degree of compaction, the authors decided to reflect these changes through the use of the VUSDFLD subroutine, which defines yield stress values (Table 1) as the criterion determining the change in material properties. This means that the input values of the parameters describing material properties that Abaqus will take for a given calculation step will depend on the determined PEEQ (equivalent plastic stress) values obtained in the previous calculation step. More details on the model used in the study can be found for instance in publication [17] in which the model had previously been described in detail.

### 2.3. Research Methodology

**Figure 4:**
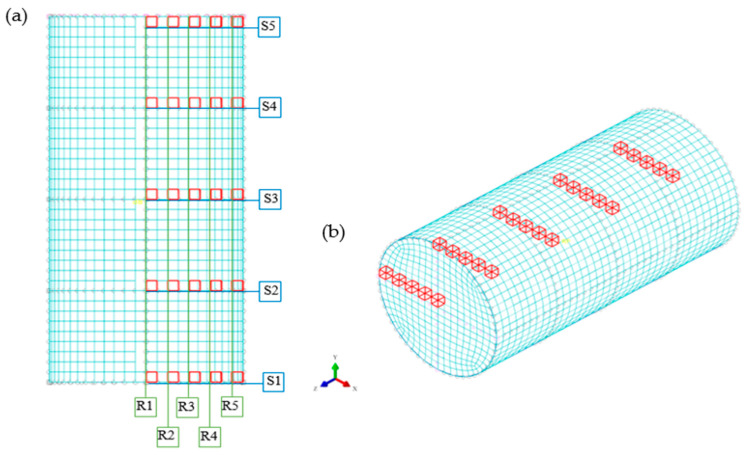
Location of the finite grid elements at which density values were read: (**a**) arrangement of elements on the cross-section of a cylindrical sample, (**b**) isometric view of a cylindrical sample.

Density distribution was determined based on the density values in the selected elements of the compacted specimen model grid. Due to the circularly symmetrical shape of the specimen, the values were read out on one-half of the specimen positioned in the ZX plane. On the selected half, 5 horizontal sections, marked with the letter S, and 5 vertical sections, marked with the letter R, were plotted. The vertical and horizontal sections were set at equal distances from each other, where the first and last vertical or horizontal radius included the extreme elements of the studied area of the model in the ZX plane. An example of the distribution of elements is illustrated in Figure 4 for a specimen with a diameter of 20 mm and an initial height of 40 mm.

The tests were carried out for geometric models with different final values of parameter *d* set within the 14 to 26 mm range, where individual parameter values were determined at 2 mm intervals. The initial mass of the specimen remained unchanged during the individual simulations and was m_0_ = 10 g. As a result, the range of the parameter *h* was limited for individual values of *d*, which was determined based on the initial value of *ρ* equal to 1062 kg/m^3^ in the DPC model used. The table with the determined values of *ρ* is shown below (Table 2). Values exceeding the acceptable density range were marked in a table with fonts with different colours. In addition, *d* values of 28 mm and 30 mm were not considered due to the insufficient number of cases in the population, i.e., less than 3.

The density variation index CV was used to compare the uniformity of DD depending on the values of the geometric parameters *d* and *h.*

In the literature, it is described as the quotient of the standard deviation by the value of the arithmetic mean $\bar{\rho }$, expressed as a percentage. The equation describing the indicated relationship is described below:

(4) $$CV=\frac{1}{\bar{\rho }}\sum \frac{\sqrt{{\left({\;\rho }_{s,\;r}-\bar{\rho }\right)}^{2}\;}}{N}\cdot 100\%.$$

In the first test, a comparison of the characteristics describing the variation of the CV value as a function of $\bar{\rho }$ was planned. The results of the study made it possible to determine the diameter value for which the best CV indication, i.e., the lowest value, occurs. In the second test, the authors set out to examine how the CV value changes for individual sections of the specimen as a function of the parameter *h* and $\bar{\rho }$, where, as previously indicated, the value of *d* was determined from an earlier test.

## 3. Results and Discussion

**Figure 5:**
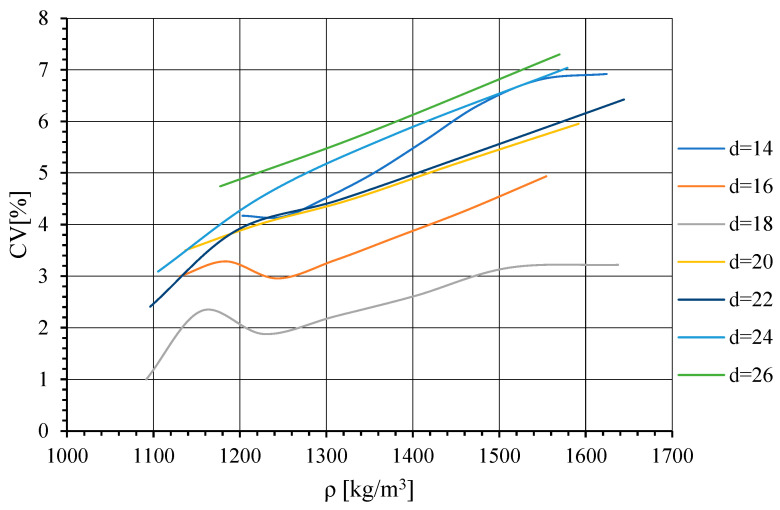
Change in CV as a function of $\bar{\rho }$ for specimens where *d* = 14–26 mm.

The results obtained in the first part of the research are presented in the graph in Figure 5. The curves represent changes in CV value as a function of $\bar{\rho }$ for specimens of the same *d* value. As has been mentioned in the numerical study methodology description, the controlled variable was the specimen height, which was directly related to the value of $\bar{\rho }$.

**Figure 6:**
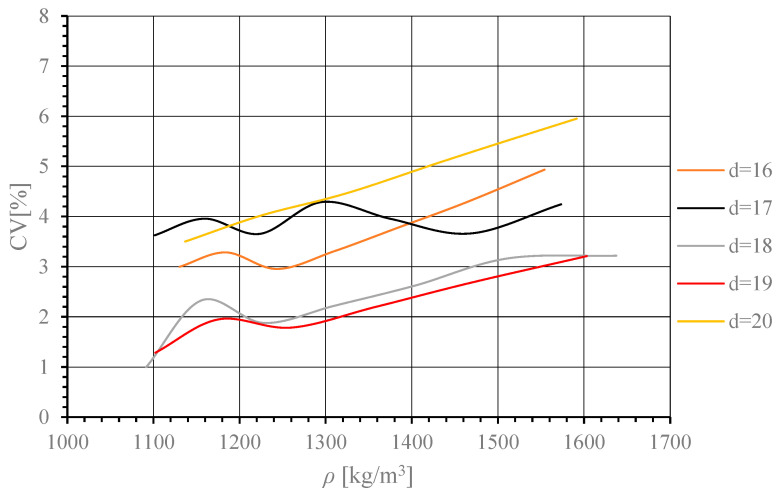
Change in CV value as a function of $\bar{\rho }$ for *d* = 16–20 mm in 1 mm increments.

As can be seen, the lowest CV value was obtained for *d* = 18 mm. Thus, it falls in the range of 16–20 mm. This being so, in the next step, the results were supplemented with numerical simulation results obtained for specimens of *d* = 17 mm and *d* = 19 mm. They are represented in Figure 6.

Thus, the lowest CV value, measured at all the test points across the specimen cross-section, was obtained for *d* = 19 mm.

Therefore, all specimens used in the second numerical experiment had the same value of *d* = 19 mm. The values of *h* and $\bar{\rho }$ were the variables in this part of the study. The parameters of the respective specimens are given in the following table (Table 3).

Significant differences in the value of CV were observed between different sections (S1, S2, S3, S4, S5) of the analysed specimens. Therefore, the following comparisons of CV values are made by section. The data in Table 4, Table 5, Table 6, Table 7 and Table 8 present the CV change as a function of *h* and $\bar{\rho }$.

Taking section S5 alone, we see that CV reached the highest level for specimens of *h* = 15 mm and $\bar{\rho }$ of 1400 kg/m^3^ and *h* = 38 mm and $\bar{\rho }$ in the range of 1550–1600 kg/m^3^. CV values fell predominantly in the range of 2–4%. Overall, the CV values varied between 0% and 6%.

Similarly to S5, and also in level S4, the lowest CV values were obtained for specimens of *h* = 15 mm and $\bar{\rho }$ = 1400 kg/m^3^ and *h* = 17 mm and $\bar{\rho }$ = 1300 kg/m^3^. For $\bar{\rho }$ = 1600 kg/m^3^, the highest CV value was obtained for specimens of *h* = 23 mm. CV values falling in the range of 2–4% prevailed. Over the whole space under analysis, CV values ranged from 0% to 6%. Comparing sections S5 and S4, we observed no significant differences in the share of CV ranges.

An additional range of maximum CV values was observed in section S3 in the specimen of *h* = 23 mm and $\bar{\rho }$ = 1600 kg/m^3^. In addition, more specimens of CV fell in the range of 4–6%. Nine specimens gave CV values in the range of 0–2%, eleven in the range of 2–4% and ten in the range of 4–6%. Only one specimen had a CV value in the range of 6–8%, specifically 6.01%. Thus, the greatest number of results fell in the range of 2–4%, with the 4–6% range following closely behind. As a result, the quality of representation of mean density is lower in this case, as compared to the S4 and S5 sections.

In the above table, we can observe an additional CV range of 8–10%. This concerned one specimen of *h* = 23 mm and $\bar{\rho }$ = 1600 kg/m^3^. In the analysed space, CV fell in the range of 0 to 2% only for one specimen of *h* = 15 mm and $\bar{\rho }$ = 1550 kg/m ^3^. For sixteen cases, CV fell in the range of 2 to 4%, and for another seven, it fell in the range of 4 to 6%. Compared to the above sections, a greater share of the 6–8% range was noted, with ten samples falling in this range. This increase, similar to the situation with section S3, indicates lower homogeneity of $\bar{\rho }$, as compared to the other sections.

In section S1, i.e., the specimen base plane, one CV value fell in the range of 10 to 12%. Three other values fell in the 0–2% range and eleven in the 4–6% range, which makes the latter range the predominant one for this plane. However, for ten cases, the CV values fell in the 8–10% range, giving an almost identical share, as was the case with section S3.

As the next step of the DD analysis of 19 mm specimens, the variation in CV values over the respective radii (R1, R2, R3, R4, R5) was checked. As in the previous step, the obtained results are presented in Table 9, Table 10, Table 11, Table 12 and Table 13, which show the change in CV values as a function of *h* and $\bar{\rho }$. In the tables, these values fall in five percentage ranges, regardless of the radii positions.

The first of these radii included elements whose one side was colinear with its rotation axis. As can be seen, this space includes five CV values falling in the 0–2% range and seventeen values in the 2–4% range. The latter of them prevails in this space. The next range of 4 to 6% includes CV values obtained for ten specimens, making it the second most significant one. The other ranges included all values obtained for three specimens. This allows us to conclude that in this radius, the value of CV fell below 4% in 77% of all cases. This indicates a high uniformity of the results in radius R1.

Radius R2 included eighteen specimens of CV falling in the 2–4% range. The above range prevailed, the same as in the case of radius R1. Eleven specimens were in the 4–6% range, with the remaining two ranges including one specimen each. In the case of the tested Division 22, the specimens had a CV value of less than 4%, which is 63% of the total area. Thus, radius R2 features a lower homogeneity section than radius R1.

Radius R3 included fourteen specimens in the 2–4% range, making it the prevalent one in the studied area. The next range of 4–6% was closely behind with thirteen specimens. The 0–2% range included four specimens of *h* = 15 and *h* = 17 mm for $\bar{\rho }$ = 1500 kg/m^3^, *h* = 21 for $\bar{\rho }$ = 1400 kg/m^3^ and *h* = 22 for $\bar{\rho }$ = 1300 kg/m^3^.

In this space, eighteen, i.e., 51% of all specimens, were in the 0–4% range, indicating lower homogeneity of specimens in radius R3, as compared to radii R1 and R2.

The other CV ranges included one specimen each.

In the R4 area, the greatest number of specimens fell in the 2–4% range, making it the prevalent one. Thirteen specimens in this group were in the 4–6% range. As in the previous radii, CV values in the 6–8% range were noted for specimens of *h* = 15 mm and $\bar{\rho }$ = 1400 kg/m^3^ and *h* = 23 mm and 1600 kg/m^3^ mean density.

CV values fell in the 0–2% range for the same specimens as in radius R3.

In total, twenty specimens, i.e., 57% of the total, fell in the 0–4% range. This indicates that in almost half of all the cases under analysis, the individual densities significantly deviated from the calculated mean density. The result was slightly better than the results. In vertical R3, however, the results were significantly worse than the results for R1 and R2.

Radius R5 included edge elements of the specimens. In this case, none of the analysed specimens fell in the outermost ranges of 0–2% and 8 to 10%; none of the test specimens were qualified. Seventeen of them fell in the 4–6% range, making it the prevailing one in this case. Fifteen other specimens had CV values ranging from 2 to 4%. To sum up, only 43% of the specimens had CV values below 4%, which indicates the worst representation of the average density of samples between the analysed verticals.

Finally, the highest CV values were noted for the element located in the lower outer corner, i.e., at the intersection of radius R5 and S1 section. Each of the examined specimens was characterised by the highest CV value.

## 4. Conclusions

Based on the analysis of the results obtained from the first numerical study, it was found that the best value for the CV index used to describe the homogeneity of the cylindrical specimens tested was obtained for specimens with a diameter of 19 mm. The obtained value can be refined in further simulations for diameters in the range from 18 to 20 mm. In the opinion of the authors, the value obtained during additional investigations will be in the range of 19–20 mm. Nonetheless, they will not significantly affect the final results of the ongoing research and production processes using dry ice.

When the results obtained in the second study were initially analysed, where the effect of specimen height *h* on its uniformity was verified, they were in line with conclusions in other scientific publications on the compaction of pulverised materials [5,23]. They confirmed that specimens with a height equal to or less than their diameter are characterised by greater uniformity.

Still, it should be pointed out that the presented results make it possible to fill the gap of knowledge in the existing literature related to the density distribution in the individual sections of the specimens. The analysis of the results focused on the distribution of density in the respective sections. As a result, it was observed that sections S5 and S4 were characterised by high homogeneity, and the results were very similar.

The second test also addressed the problem of uniformity of density depending on the location of the analysed vertical radius on the specimen, i.e., its distance from the centre of rotation of the cylindrical specimen. The results demonstrated that, as the distance from the specimen axis increases, the homogeneity of the material decreases, where the values obtained for radii R1 and R2, similarly as in the case of sections L5 and L4, were very similar, with high homogeneity noted. The results obtained also indicate that, in the case of a compressed pulverised material such as dry ice, the surface density measurement methods will be characterised by a large discrepancy with respect to the average density of the compacted material.

With a view to improving the quality of the dry ice pellets obtained and to reducing the energy intensity of the processes used to produce them, it is proposed that research is undertaken on the development of new machines suited for the production of dry ice pellets with a diameter in the range of 18–20 mm. No machines for the extrusion of pellets within the indicated diameter range have been identified on the market; hence, further numerical studies are planned to help develop a machine that can produce pellets of the desired diameter.

As indicated in the article, the studies utilised the DPC model, calibrated based on laboratory studies using bulk dry ice with a particle size of approximately 100 μm. However, it should be noted that the literature does not highlight any studies concerning the effect of loose dry ice particle size on its extrusion process. Such material can be obtained by using the cryomilling technique, which enables the milling of materials under reduced temperature conditions, as described by Guan et al. 2021 and Maines et al. 2024 [24,25]. Based on this, it is possible to deepen the presented research, taking into account the influence of the dry ice fraction size on the parameters of the thickening process.

## Figures and Tables

**Table 1:** Values of DPC model parameters [17].

Material Cohesion [MPa]	Angle of Friction [deg]	Cap Eccentricity [Pa]	Init Yld Surf Pos [-]	Yield Stress [MPa]	Vol Plas Strain [-]	Young’s Modulus [MPa]	Poisson’s Ratio [-]
1.07	25.92	0.68	0.02	1.24	0	136.94	0.023
1.75	21.46	0.76	0.02	1.57	0.048	194.18	0.059
2.22	20.51	0.78	0.02	1.92	0.095	251.42	0.102
2.53	20.59	0.79	0.02	3.20	0.139	308.66	0.150
2.73	21.05	0.80	0.02	4.50	0.182	365.9	0.200
2.85	21.59	0.81	0.02	6.54	0.223	423.14	0.249
2.93	22.08	0.83	0.02	7.92	0.262	480.38	0.295
3.00	22.43	0.84	0.02	10.25	0.300	537.62	0.335
3.07	22.61	0.87	0.02	13.1	0.336	594.86	0.370
3.14	22.64	0.90	0.02	16.57	0.371	652.1	0.399
3.23	22.60	0.93	0.02	21.51	0.405	709.34	0.422
3.32	22.63	0.98	0.02	27.22	0.438	766.58	0.441
3.40	22.88	1.03	0.02	32.06	0.470	823.82	0.456

**Table 2:** The final value *ρ* [kg/m^3^] for the cylindrical specimen with a total mass m_0_ = 10 g, for various *d* and *h* values.

*h* [mm]	*d* [mm]						
	14	16	18	20	22	24	26
**12**	5413	4145	3275	2653	2192	1842	1570
**14**	4640	3553	2807	2274	1879	1579	1345
**16**	4060	3108	2456	1989	1644	1382	1177
**18**	3609	2763	2183	1768	1461	1228	1046
**20**	3248	2487	1965	1592	1315	1105	942
**22**	2953	2261	1786	1447	1196	1005	856
**24**	2707	2072	1637	1326	1096	921	785
**26**	2499	1913	1511	1224	1012	850	724
**28**	2320	1776	1403	1137	940	789	673
**30**	2165	1658	1310	1061	877	737	628
**32**	2030	1554	1228	995	822	691	589
**34**	1911	1463	1156	936	774	650	554
**36**	1804	1382	1092	884	731	614	523
**38**	1710	1309	1034	838	692	582	496
**40**	1624	1243	982	796	658	553	471
**42**	1547	1184	936	758	626	526	448
**44**	1476	1130	893	723	598	502	428
**46**	1412	1081	854	692	572	481	409
**48**	1353	1036	819	663	548	461	392
**50**	1299	995	786	637	526	442	377

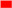
—value above the maximum DI density value, equal to the limit of 1650 kg/m^3^. 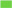
—value below the bulk density DI, equal to the limit of 550 kg/m^3^. 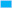
—DI density value, below the initial value in the numerical model, equal to the limit of 1062 kg/m^3^.

**Table 3:** Parameters of cylindrical specimens used in the second numerical experiment.

*h* [mm]	$\bar{\rho }$ [kg/m^3^]
15; 19; 21; 22; 29	1300; 1400; 1500; 1550; 1600

**Table 4:** Change in CV [%] value as a function of *h* and $\bar{\rho }$ in S5 section, *d* = 19 mm specimen.

*h* [mm]	$\bar{\rho }$ [kg/m^3^]				
	1300	1400	1500	1550	1600
15	2.2	4.7	3.1	1.2	3.4
17	5.4	2.1	1.7	0.8	2.7
19	4.9	0.9	3.4	1.2	2.2
21	1.8	1.4	2.2	2.8	1.5
23	0.6	3.9	3.2	2.2	4.9
29	2.5	2.0	1.5	4.1	1.0
38	1.7	3.0	3.0	4.0	4.2

**Table 5:** Change in CV [%] as a function of *h* and $\bar{\rho }$ in section S4, specimen of *d* = 19 mm.

*h* [mm]	$\bar{\rho }$ [kg/m^3^]				
	1300	1400	1500	1550	1600
15	2.6	5.2	2.2	1.2	4.2
17	4.9	2.6	1.0	0.8	3.4
19	4.4	0.8	3.9	0.7	2.6
21	2.1	1.0	2.5	2.2	1.2
23	0.5	4.2	2.5	2.4	5.4
29	2.8	1.5	1.4	3.4	0.9
38	1.3	2.5	2.4	3.3	3.4

**Table 6:** Change in CV [%] value as a function of *h* and $\bar{\rho }$ in S5 section, *d* = 19 mm specimen.

*h* [mm]	$\bar{\rho }$ [kg/m^3^]				
	1300	1400	1500	1550	1600
15	3.3	6.0	0.6	2.6	5.6
17	3.7	3.7	0.7	2.1	5.2
19	3.1	2.1	5.5	1.8	4.8
21	3.7	0.7	4.5	0.5	3.6
23	1.8	5.9	0.3	5.1	8.1
29	4.5	1.1	4.4	0.8	3.9
38	1.5	1.1	2.5	2.0	1.9

**Table 7:** Change in CV [%] as a function of *h* and $\bar{\rho }$ in section S2, specimen of *d* = 19 mm.

*h* [mm]	$\bar{\rho }$ [kg/m^3^]				
	1300	1400	1500	1550	1600
15	3.9	6.7	0.7	3.9	6.9
17	2.6	4.7	2.1	3.8	6.8
19	2.0	3.4	6.8	3.7	6.7
21	4.8	2.3	6.0	2.8	5.9
23	3.0	7.1	2.4	7.2	10.1
29	5.7	3.2	6.6	3.8	6.8
38	3.5	3.9	5.7	5.7	6.1

**Table 8:** Change in CV [%] as a function of *h* and $\bar{\rho }$ in S5 section, *d* = 19 mm specimen.

*h* [mm]	$\bar{\rho }$ [kg/m^3^]				
	1300	1400	1500	1550	1600
15	4.5	7.5	2.0	5.2	8.2
17	1.7	5.7	3.4	5.2	8.2
19	1.3	4.5	8.0	5.3	8.2
21	5.8	3.6	7.3	4.7	7.6
23	4.1	8.2	4.2	8.8	11.6
29	7.0	4.8	8.4	6.2	9.1
38	5.1	6.0	8.1	8.5	9.2

**Table 9:** Change in CV [%] as a function of *h* and $\bar{\rho }$ in radius R1 of *d* = 19 mm specimen.

*h* [mm]	$\bar{\rho }$ [kg/m^3^]				
	1300	1400	1500	1550	1600
15	3.3	6.0	1.1	2.6	5.7
17	3.6	3.8	1.2	2.2	5.3
19	3.1	2.2	5.6	2.1	4.9
21	3.7	1.4	4.5	2.0	3.8
23	1.9	5.9	1.9	5.3	8.2
29	4.7	2.1	4.5	3.0	4.0
38	2.2	2.7	3.6	4.0	4.1

**Table 10:** Change in CV [%] as a function of *h* and $\bar{\rho }$ in radius R2 of *d* = 19 mm specimen.

*h* [mm]	$\bar{\rho }$ [kg/m^3^]				
	1300	1400	1500	1550	1600
15	3.4	6.1	1.2	2.8	5.9
17	3.5	3.9	1.2	2.3	5.4
19	3.0	2.3	5.6	2.1	5.0
21	3.7	1.5	4.5	2.1	3.8
23	1.9	5.9	2.1	5.2	8.1
29	4.6	2.2	4.4	3.2	4.0
38	2.4	3.0	4.0	4.3	4.5

**Table 11:** Change in CV [%] as a function of *h* and $\bar{\rho }$ in radius R3 of *d* = 19 mm specimen.

*h* [mm]	$\bar{\rho }$ [kg/m^3^]				
	1300	1400	1500	1550	1600
15	3.4	6.2	1.4	2.8	5.9
17	3.5	3.9	1.5	2.3	5.4
19	3.0	2.3	5.6	2.2	5.0
21	3.7	1.6	4.5	2.3	3.8
23	1.9	5.9	2.2	5.2	8.2
29	4.6	2.3	4.4	3.3	4.0
38	2.4	3.1	4.0	4.4	4.6

**Table 12:** Change in CV [%] as a function of *h* and $\bar{\rho }$ in radius R4 of *d* = 19 mm specimen.

*h* [mm]	$\bar{\rho }$ [kg/m^3^]				
	1300	1400	1500	1550	1600
15	3.3	6.1	1.9	2.6	5.6
17	3.6	3.8	1.9	2.4	5.4
19	3.0	2.2	5.5	2.5	4.9
21	3.6	1.8	4.5	2.5	3.9
23	1.8	5.9	2.5	5.1	8.1
29	4.5	2.4	4.3	3.6	4.1
38	2.5	3.1	4.2	4.6	4.8

**Table 13:** Change in CV [%] as a function of *h* and $\bar{\rho }$ in radius R5 of *d* = 19 mm specimen.

*h* [mm]	$\bar{\rho }$				
	1300	1400	1500	1550	1600
15	3.1	5.9	3.0	3.3	5.3
17	4.0	3.5	3.0	3.5	4.9
19	3.7	2.7	5.2	3.8	4.7
21	3.4	2.8	4.3	4.0	4.5
23	2.5	5.6	3.9	5.1	7.6
29	4.3	3.7	4.8	5.2	5.6
38	3.6	4.6	6.0	6.4	6.8

## Data Availability

The original contributions presented in the study are included in the article, further inquiries can be directed to the corresponding author.
